# Manipulation of the Complement System for Benefit in Sepsis

**DOI:** 10.1155/2012/427607

**Published:** 2012-03-05

**Authors:** Peter A. Ward, Ren-Feng Guo, Niels C. Riedemann

**Affiliations:** ^1^Department of Pathology, University of Michigan Medical School, 1301 Catherine Road, P.O. Box 5602, Ann Arbor, MI 48109-5602, USA; ^2^Department of Anesthesiology and Intensive Care Medicine, University Hospital Jena, 07747 Jena, Germany

## Abstract

There is evidence in sepsis, both in rodents and in humans, that activation of the complement system results in excessive production of C5a, which triggers a series of events leading to septic shock, multiorgan failure, and lethality. In rodents following cecal ligation and puncture (CLP), which induces polymicrobial sepsis, in vivo blockade of C5a using neutralizing antibodies dramatically improved survival, reduced apoptosis of lymphoid cells, and attenuated the ensuing coagulopathy. Based on these data, it seems reasonable to consider therapeutic blockade of C5a in humans entering into sepsis and septic shock. Strategies for the development of such an antibody for use in humans are presented.

## 1. Introduction

Sepsis and septic shock are very challenging medical problems for which specific interventional therapy is currently extremely limited. The resulting outcome is substantial lethality. In both experimental (polymicrobial) sepsis and human sepsis/septic shock there is evidence for robust activation of the complement system, resulting in release of extremely strong proinflammatory products such as C5a, an anaphylatoxin that reacts with its receptors (C5aR, C5L2) on phagocytes (neutrophils, macrophages) and on a variety of organs to trigger numerous biological responses (enzyme release, chemotaxis, respiratory burst resulting in production of O_2_^•^ and H_2_O_2_, and other responses) [1]. The complement system is a key component of the innate immune system, activation of which results in production of C3b (from C3), which is a key opsonic factor reactive with receptors on phagocytes to promote internalization of bacteria and their subsequent destruction. An activation product of the distal complement pathway that reflects the innate immune system is the membrane attack complex (C5b-9) which causes lysis of Gram-negative bacteria. C3a and C5a anaphylatoxins are small cleavage products from C3 and C5 and possess proinflammatory activities, especially C5a. As sepsis development proceeds, there is a burst of C5a production which results in excessive activation of phagocytic cells, often leading to paralysis of MAPK signaling pathways [2]. Also part of the response to sepsis is upregulation of C5aR on a variety of nonphagocytic cells in liver, spleen, kidneys, and lungs [3], which, when interactive with C5a, is associated with multiorgan failure. In this paper, we will review evidence, mostly from our own laboratories, regarding the ability of neutralizing antibody to C5a to dramatically reduce lethality in septic rodents as well as reducing apoptosis of lymphoid cells (leading to immunodeficiency) and the coagulopathy of sepsis. We will also discuss issues regarding the development of antibodies to human C5a that might mitigate the complications of sepsis. 

## 2. Complement Pathway Activation

**Figure 1:**
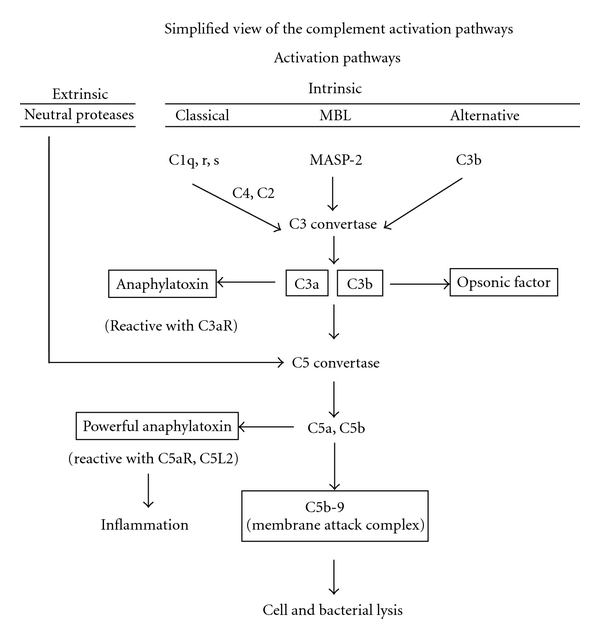
Simplified view of intrinsic and extrinsic pathways of complement activation. Boxes show major activation products of complement activation and the biological consequences of these products.

Figure 1 is a simplified version of the various pathways of complement activation. 

The traditional pathways include the classical pathway (which sequentially activates C1, C4, and C2 to produce the C3 convertase) and the *mannan binding lectin (MBL)* pathway (also known as mannan binding protein, MBP) in which MBP binds to mannose-rich glycans on bacterial surfaces. This leads to activation, depending on the species, of mannan-binding lectin-associated serine proteases [1–3] (MASP), the first two proteases being similar to C1r and C1s of the classical pathway. The end result is cleavage of C4 and C2 to form C4b2a, the C3 convertase that will then generate C3a and C3b. The third pathway of complement activation is related to the constant, spontaneous hydrolysis of C3, resulting in formation of C3b, which then triggers complement activation. An extrinsic pathway of complement activation relates to direct activation (cleavage) of C5 by cell/tissue/plasma neutral proteases (such as thrombin and proteases released from neutrophils and macrophages), resulting in formation of C5a and C5b. Figure 1 also shows (in boxes) the complement activation products that are relevant in sepsis. These are C5a and C3b, the latter being a major opsonic factor reactive with bacteria to promote their phagocytosis. It is clear that C3b is a major product facilitating the protective effects of complement in the innate immune system, providing a protective shield against infections agents. Targeting C3 or its activation products especially C3b in the setting of sepsis, unless very carefully regulated, has the potential to depress the opsonic system, which is vital for the in vivo response to bacteria in the setting of sepsis. C5a is a very powerful phlogistic product of the complement system which, in the setting of sepsis and when produced in excessive quantities, can result in catastrophic outcomes, which will be discussed below. The ability to dampen the effects of C5a includes its in vivo neutralization or blockade in vivo of its two receptors, C5aR and C5L2. Finally, production of the membrane attack complex (C5b-9) may play a protective role in the setting of sepsis, since C5b-9 has the ability to engage in lysis of Gram-negative bacteria. For this reason, the use of antibodies to deplete C5 in vivo will depress C5a generation, reducing the production of C5b-9, which is an undesirable outcome (described below).

## 3. Survival after CLP Based on Mouse Genotypes

We have employed CLP in C57BL/6 young adult males (25 gm) and have used various genotypes, as described in Table 1. 

Two grades of CLP, as recently described in detail [4] have been employed. High-grade sepsis (75% of cecum ligated) in Wt mice resulted in no survivals by the end of day 3 [4]. Use of similar septic conditions in C5aR^−/−^ or C5L2^−/−^ mice resulted in survival rates on day 3 of 85% and 80%, respectively, suggesting that engagement of both C5a receptors, perhaps operating in sequence, results in highly adverse outcomes, as seen in Wt mice. Data suggesting sequential engagement of the two C5a receptors in the setting of sepsis indicated that the bulk of the proinflammatory mediators appearing in plasma 24 hr after CLP were reduced by ≥80% in the k. o. mice [5]. The fact that absence of either receptor resulted in suppression of mediator production far above the 50% level suggests that there may be some type of collaborative interaction between C5aR and C5L2 during development of sepsis. When intermediate grade sepsis (50% of cecum ligated) was employed, survival rates in Wt, C3^−/−^ or C5^−/−^ mice by day 7 were 31%, 5% and 15%, respectively. In the case of C5aR^−/−^ or C5L2^−/−^ mice, survival rates were 80% and 100%, respectively, correlating with results in mice with high grade sepsis (top of Table 1). The high lethality (95%) in C3^−/−^ mice may be due to the inability to generate the opsonic factor, C3b. The fact that C5^−/−^ mice did worse than the Wt mice even though no C5a formation occurs could be related to inability to generate MAC (C5b-9) which has lytic activity for Gram-negative bacteria (see Section 7.3). Since we previously found that C3^−/−^ mice, at least in the setting of acute lung injury, can generate C5a via the role of thrombin functioning as a C5 convertase [6], this suggests that C3^−/−^ mice can still generate some C5a, which complicates interpretation of data. The parallel data of high survival in C5aR^−/−^ or C5L2^−/−^ mice in both high grade and intermediate grade sepsis is consistent with the idea that both receptors act, perhaps simultaneously or sequentially, to cause adverse consequences after onset of CLP.

## 4. Use of Blocking Antibodies to C5a in Animal Sepsis

Experimental studies in the 1980s involving *monkeys* and employing a rabbit anti-human polyclonal antibody to C5a indicated that blockade of C5a by such antibodies could significantly attenuate live *Escherichia coli*-induced septic shock and accompanying acute respiratory distress syndrome (ARDS) [7] and that such antibodies could significantly lower the C5a levels in monkey blood [8]. In a model of severe sepsis in *pigs* due to infusion of live *E. coli,* administration of a neutralizing monoclonal anti-pig C5a antibody (not cross reactive with C5) led to significantly decreased levels (75%) of IL-6 [9]. In the same model, this antibody also was demonstrated to significantly improve the oxygen utilization during severe sepsis [10]. In studies in *rats* it was suggested that LPS-induced septic shock was associated with increased C5a levels in blood and that equivalent septic shock features could be mimicked by infusion of C5a. Blockade of C5a in this model with F(ab′)2 fragments of rabbit anti-rat C5a significantly attenuated LPS-induced responses [11].

In recent work, the Michigan group has demonstrated in rats following cecal ligation and puncture-induced sepsis that blockade of C5a with polyclonal rabbit anti-rat antibodies significantly improved survival in septic rats and lowered the intensity of the inflammatory responses (plasma/serum/cytokines/chemokines) [12]. Different anti-C5a peptide antibodies (affinity purified) to rat C5a were then investigated in the same model. Antibodies were directed to different regions of the C5a molecule, assuming that blockade of certain regions of C5a would be expected to provide the highest protective effects after CLP [13]. Certain of these antibodies were demonstrated to significantly reduce the occurrence of multiorgan failure [14]. Details of these studies are discussed below. The Michigan group also demonstrated that polyclonal blocking antibodies directed against rat C5a and cross-reacting with mouse C5a significantly improved survival in CLP-mice and that such improvement in survival was comparable to the improvement that could be achieved by employing a specific polyclonal anti-mouse C5a antibody to C5aR in the same model [3]. In a recent study such results were confirmed by employing rabbit polyclonal anti-mouse C5a antibody [5].

In addition to the data described above, numerous publications describe the use of polyclonal and monoclonal anti-C5a antibodies and their efficacies in models of inflammation such as ischemia/reperfusion injury, renal disease, graft rejection, malaria, rheumatoid arthritis, infectious bowel disease, inflammatory lung disease, and lupus-like autoimmune diseases. using various animal species (reviewed, [15]).

Table 2 demonstrates our studies dealing with the abilities of antigen affinity purified rabbit IgG preparations to protect CLP rats (young adult 300 gm male Sprague-Dawley) as a function of the antibody preparation and the time after CLP of intravenous infusion of 600 *μ*g IgG [13]. 

When given just before CLP, antibody to the M region of rat C5a (amino acid residues 17–36) resulted in 90% survival, while antibody to the C region (residues 58–77) provided equivalent survival (85%), which is contrast to preimmune rabbit IgG, in which only 30% survival ensued. When the IgG preparations were infused 6 hr after onset of CLP, antibodies to the M and C regions of rat C5a still provided good protection, resulting in 70% and 60% survival, respectively, while infusion of preimmune IgG at the same time after CLP resulted in only 35% survival. When antibody infusions were delayed until 12 hr after CLP, antibodies to the M and C regions still had impressive protective effects, providing 50% and 40% survival, respectively, while infusion of preimmune IgG resulted in only 17% survival. Antibodies that were generated and were reactive with the N terminal region of rat C5a (residues 1–16) were ineffective in producing beneficial effects in CLP rats (data not shown). Collectively, these data indicate that neutralizing polyclonal antibodies to two different regions of rat C5a have significantly protective effects by enhancing survival after CLP in rats. Furthermore, the antibodies still had protective effects when the infusion was delayed for 6 or 12 hr after CLP. This gives hope that, in septic humans, infusion of C5a neutralizing antibodies may be protective even if used in the later phases of sepsis.

## 5. Ability of Mouse Monoclonal Antibodies to Rat C5a to Improve Survival after CLP

Preliminary studies have been done using mouse mAbs to rat C5a in order to determine if mAbs would also improve survival in CLP rats. These antibodies (IgG_2_) were shown by plasma resonance measurements to be of high affinity, although in these studies the epitopes on C5a that were reactive with the mAb were not determined. As shown in Table 3, using high grade CLP, 1000 *μ*g normal IgG_2_ resulted in a 5 day survival of only 10%, whereas infusion of 1,000 *μ*g or 400 *μ*g of mAb resulted in 70% and 65% survival, respectively.

## 6. Role of C5a in Apoptosis of Thymocytes

Apoptosis of T and B cells has been extensively described in CLP mice [16, 17] and in septic humans [18]. These events occur very early in the setting of sepsis in humans, often in the first 12 hr after hospital admission and are thought to be responsible for early development of immunosuppression in humans and also in rodents. Treatment of mice with caspase inhibitors *before* onset of sepsis enhanced survival [19]. We have evaluated rats after onset of CLP and found over the first 48 hr substantial loss of thymic mass (by 59%) (data not shown). Treatment of these animals before CLP with neutralizing antibody to rat C5a reduced the loss of thymic mass to a value of 26% [20], allowing the interpretation that C5a is in some manner linked to induction of thymocyte apoptosis. In other studies we found a surprising result, namely, that 3 hr after CLP thymocytes showed a 65% increased binding of ^125^I-C5a, associated with an early and rapid increase in mRNA for C5aR [21]. In addition, in vitro addition of C5a to thymocytes obtained from CLP rats resulted in increased binding of Annexin V, which correlated with caspase activation and apoptosis [21].

Caspase content was measured in rat thymocytes employing fluorochrome peptide substrates specific for individual caspases, using thymocytes obtained from rats subjected to CLP 12 hr earlier. There were substantial elevations in caspases 3 (nearly 4-fold), 6 (3-fold), and 9 (almost 3-fold) in rats given 400 *μ*g preimmune rabbit IgG, while caspase 8 showed no elevation (Table 4). 

In a companion set of CLP rats given 400 *μ*g neutralizing antibody to C5a, the fold increases in caspases 3, 6, and 9 were considerably reduced. Correlating with the data in Table 4, thymocytes were obtained 12 hr after CLP.  Table 5 describes Annexin V binding to thymocytes from sham rats (laparotomy without cecal ligation and puncture) and from rats subjected to CLP following intravenous infusions of 400 *μ*g preimmune rabbit IgG or 400 *μ*g affinity purified C5a neutralizing rabbit IgG. In vitro binding of Annexin V to thymocytes was quantitated by flow cytometry. As is evident in Table 5, only 4% of thymocytes from sham rats bound Annexin V, whereas 12 hr after CLP in rats pretreated in vivo with preimmune IgG, 29% thymocytes bound Annexin V. 

In striking contrast, the binding of Annexin V to thymocytes from CLP rats treated with neutralizing anti-C5a antibody fell to 7%. Collectively, these data indicate that thymocyte apoptosis can be linked to C5a, C5aR and early elevation in C5a binding to thymocyte C5aR following CLP. It seems likely that events of this type may apply in general to the lymphoid system as a whole in the setting of sepsis.

## 7. Developing Anti-C5a Antibodies for Use in Humans

### 7.1. General Aspects

There is abundant literature from various scientific groups detailing the important role of C5a in acute inflammatory diseases and especially in sepsis. As indicated above, this evidence goes back many years, to the 1980s [22, 23]. In the meantime, a much more in-depth understanding about the molecular and cellular mechanisms involved in C5a-induced harmful in vivo effects in sepsis is now available. It is therefore reasonable to ask the question why no effective compounds targeting C5a or its receptors have yet been developed by pharmaceutical companies that display a growing hunger for potential block-buster drugs by targeting key players that are driving disease progression in humans. There is no easy answer to this question, but the following reasons may have contributed. (1) The complement system has long been perceived as being dangerous to “play” with. Such reluctance to tamper with complement stands in striking contrast to the efforts over the past 50 years to suppress adaptive immunity as related to allografts in humans, knowing full well that immunosuppression, if excessive, can lead to catastrophic outcomes in humans. As a major participant in the innate immune system, complement has been considered to be sacrosanct. Many clinicians and scientists have been aware of the role of complement in its antimicrobial role exerted through the terminal membrane attack complex [5] and the important opsonic function of C3b leading to a stimulation of the immune response, but information did not generally extend beyond these areas. Due to the complexity of the complement system and its obvious importance in both innate immunity and adaptive immunity, there seems to have been an instinctive reluctance to develop drugs that block components of the complement system. In addition, certain drugs targeting the upstream complement components such as C1 inhibitor did not demonstrate convincing therapeutic potential in humans [24]. (2) C5a is an especially difficult target for drug development. It can be produced in large amounts in the plasma within minutes, but yet it is elusive and difficult to measure because of the various mechanisms for its removal from the blood stream (binding to receptors on blood leukocytes, rapid clearance in the kidneys, etc). In addition, C5a is a small molecule that is cleaved from of its parent molecule, C5, and it is clear that many antibodies binding to C5a will also bind to C5. The latter observation implies a potential for such antibodies to interfere with C5b-induced formation of the MAC. Loss of C5 function in sepsis would be a highly undesirable outcome since MAC causes lysis of Gram-negative bacteria.

### 7.2. Selecting an Anti-C5a Approach versus an Anti-C5aR Approach

Given the above-mentioned concerns, one might speculate whether it might be better and easier to develop anti-C5aR antibodies. From a technical perspective, this appears to be true, and there is evidence in experimental sepsis that anti-C5aR and even also anti-C5L2 antibodies exert beneficial effects [5]. However, one needs to consider that there is an abundant amount of these receptors on almost all cell types. One neutrophil alone is estimated to display approximately 200,000 C5a binding receptors on its surface [25]. In addition, we were able to demonstrate that C5aR is induced in various organs and cell types during the development of sepsis (CLP) in rodents [3]. C5a binds very rapidly to these receptors and, in order to achieve a complete blockade of C5a-induced effects, it would likely be necessary to block virtually all available receptors. With this in mind, one might speculate if it would be more efficient to block C5a directly rather than C5aR. On the other hand, various research reports have demonstrated highly significant beneficial effects for outcome during experimental sepsis in rodents using either blocking antibodies or small chemical inhibitors to C5aR. It should be mentioned that Novo Nordisc has developed the first anti-human C5aR antibody for use in clinical phase I trials, for application in rheumatoid arthritis (according to the company's press release). As far as our understanding currently goes, it remains to be determined in future clinical trials, using blockade of C5aR or C5a directly, which target might be more beneficial in the setting of inflammatory diseases, including sepsis.

### 7.3. Blocking C5—An Approach That Has Made It to the Market with Great Success but May Be with Associated Risks

From a technical perspective, blocking C5 with an antibody is a much easier strategy than a blocking antibody to C5a that often also reacts with C5. The C5 molecule is present in large amounts in the serum. However, due to the size of C5a and C5b, antibodies which bind to C5 and effectively block formation of C5a would do so primarily by depleting C5 or preventing cleavage of C5 to C5a and C5b. Such antibodies would therefore compromise the function of C5b and the related MAC as well as leading to susceptibility of treated patients towards bacterial and other microorganism-driven infections. However, for life-threatening diseases that are primarily driven by the action of MAC, such antibodies represent a valuable therapeutic strategy. The high risk for developing bacterial infections might be counteracted by immunization of patients before the use of an antibody that depletes C5.

It is not surprising that an effective humanized anti-C5 antibody has been successfully developed into a marketed product (Soliris: Eculizumab by Alexion Pharmaceuticals). This development has provided great hope for researchers and biotech and pharmaceutical companies that are trying to prove that great potential lies in blocking selected targets within the complement system in the setting of various diseases. Soliris is currently approved for use in paroxysmal nocturnal hemoglobinuria (PNH), a disease which is driven by complement-induced lysis of red blood cells and other cells within the immune system, due to a genetic defect leading to a lack of an anchor protein which holds a cell bound complement inhibitor on the cell surface. Soliris has displayed excellent lifesaving therapeutic potential in PNH patients. Recent reports suggest that treatment of such patients with Soliris appears to result in relatively normal life expectancy [26]. Because of the antibody amount needed for an effective C5 blockade (normal serum levels up to 400 nM), and due to high development costs of Soliris as well as PNH being a small market, the average yearly treatment costs exceed $300,000 (USD) per patient.

Soliris is about to receive marketing authorization for use in atypical hemolytic uremic syndrome (aHUS) in the USA within the next few months. aHUS is a disease in which the role of MAC versus C5a is not understood in detail. However, Alexion has been also conducting clinical phase II trials using Soliris in other acute inflammatory diseases, such as antibody-mediated transplant graft rejection following kidney transplantation, in which it might be speculated whether C5a is the disease-driving key element rather than the MAC. If this were true, a C5a specific blocking antibody which would not interfere with C5b and MAC formation would be expected to be a more desirable strategy for treatment. A high efficacy antibody could be used at lower concentrations and such antibody application might not display the associated risks of infection. This would be of great importance for immune-compromised patients such as transplant recipients.

Due to the associated risks of Soliris related to blocking MAC formation, patients receiving this antibody need to be immunized against meningococcal infections to prevent from meningococcal-induced sepsis. It is well known that humans with absence of C5 have susceptibility to neisserial infections. It is therefore implied that a C5 blocking antibody should not be used in acute inflammatory diseases which are microbial driven, such as sepsis and others. A recent publication from the Ward laboratories [27] used mice depleted of C6 by an antibody, which would be expected to largely prevent MAC formation in the setting of CLP. Blood CFUs were evaluated 24 hr after CLP. Wt mice had approximately 2×10^5^ CFUs/mL, C3^−/−^ mice had a 4-fold increase, and C5^−/−^ CLP mice had a 400-fold increase in blood CFUs. C6 “depleted” CLP mice had CFU levels that were 15-fold higher than complement-intact mice. The fold increase in these mice was probably much lower compared to C5^−/−^ because of incomplete depletion of C6. These data suggest that the MAC plays an important protective role in the setting of CLP in mice.

### 7.4. Technical Aspects and Requirements for Development of Effective Anti-C5a Antibodies

Obviously, designing an effective antibody for blocking C5a for use in humans appears to be rather difficult since no such molecule has yet been developed for clinical testing in patients (as far as being currently publically disclosed). From a purely technological standpoint, generating antibodies can be easily achieved using various different platforms for obtaining standard molecules following immunization of animals (polyclonal or monoclonal antibodies). Humanization of mouse antibodies can be done in the case of monoclonal antibodies or even fully human monoclonal molecules can be derived from phage display libraries and also from genetically modified animals that generate human molecules. There is great debate in the literature about the questions of which of these groups of antibodies may have most favorable biological features. However, one consensus appears to be that every molecule is unique and will contain unique properties which may or may not be typical for its group. In the end, one question always remains to be answered: how effectively does the molecule do what it is assumed to do: block C5a biological effects in vivo?

## 8. Features of Desirable Antibodies

From a researcher's perspective the following requirements should be met for a suitable anti-C5a monoclonal antibody candidate:

- high biological blocking efficacy;
- high C5a selectivity—no interference of C5 and C5b;
- fast, robust, and strong binding to C5a;
- no binding to other targets (cell surfaces, receptors, etc.);
- low immunogenicity (low anti-drug antibody formation);
- desirable half-life in serum;
- desirable biological activity over time.

### 8.1. High Blocking Efficacy

 This requirement is important because C5a can be produced in large amounts—theoretically up to the total amount of serum C5 if every molecule C5 would be cleaved (400 nM). Therefore, strong biological efficacy of an antibody would guarantee a lower total amount of antibody to be needed in acute settings such as sepsis. Since low amounts of C5a (10 nM) are capable of significantly boosting immunological responses in vitro, it would be important to be able to completely suppress C5a-induced biological effects. Ultimately, high antibody efficacy could also result in lower costs of a product in the market.

### 8.2. High C5a Selectivity

As outlined in detail above, this feature would be needed in order to guarantee that C5b and MAC formation would be undisturbed such that effective microbial removal through complement-induced lysis would be possible. However, selectivity may be the most difficult feature to be achieved because of the following fact. According to crystal structure analysis in a recent work by Fredslund et al. [28] it becomes clear that binding epitopes of C5a, which are most important for the interaction with its receptors (C5aR and C5L2), are facing the outside within the parent molecule C5 (before cleavage). Biologically effective blocking antibodies targeting C5a must bind to such epitopes in order to hinder C5a from interacting with its receptors. Thus, such molecules are highly likely to bind not only to C5a but also to C5. Therefore, finding a functional neoepitope on C5a but not on C5 is the first challenge to overcome for the development of such a therapeutic antibody.

### 8.3. Rapid Binding

The binding of C5a to its receptors occurs rapidly, leading to activation of C5a receptors and in case of C5aR to the internalization of the C5a•C5aR complex. Therefore, it is expected that a useful anti-C5a mAb should have a binding constant to its target (C5a) lower than the one of C5a to its receptors in order to assure that antibody present in serum would be binding newly generated C5a before the C5a would react with its receptors.

### 8.4. No Binding to Other Targets/Structures

 It has become increasingly clear that unwanted features of antibodies could result in serious consequences [29]. Ultimately, an antibody binding to a circulating protein should ideally not bind to surfaces, receptors or other proteins that could interact with cell biological processes and trigger a biological response (“agonist antibodies”). Therefore, an anti-C5a antibody under development must be screened for unwanted cytotoxic/immunotoxic and other effects.

### 8.5. Low Immunogenicity

Formation of anti-drug antibodies is a well-known problem, even if not predictable based on the structure of the monoclonal antibody, the outcome of which could limit biological efficacy and availability of monoclonal antibodies. This problem would be most relevant when a drug is intended for long term use, requiring multiple injections. Since long term administration of antibody is not likely in the case of sepsis, it needs to be kept in mind that international guidelines require the development of validated tests demonstrating the extent of anti-drug antibody formation in humans. Formation of such antibodies must be further evaluated with regard to impacts on the biological efficacy of the antibody.

### 8.6. Desirable Half-Life and Biological Activity in Serum

Complement activation should be ongoing not only during the initial phase of sepsis development but probably also during the course of sepsis. It is important to generate antibodies that will be available and biologically active during a time span of at least several days. This feature will guarantee protection from C5a induced harmful effects over a time exceeding the initial hours of sepsis development. Presently, various possibilities exist to alter antibody clearance through induced changes in the antibody on a molecular level. Different IgG structures (IgG1, IgG2, IgG4, etc.) are commonly used to preselect certain desired half-life features and also some associated immunological reactions (e.g., Fc-mediated complement activation, etc.).

### 8.7. Safety and Therapeutic Rational for Use of Anti-C5a Antibodies in Patients with Sepsis

Since the Soliris product, anti-C5 antibody, which also prohibits generation of C5a because of C5 depletion or blockade, has been approved and because the available data in the development program indicate a very good safety and tolerability profile, one may assume that an anti-C5a antibody, being more specific and leaving C5b and MAC formation untouched, should be safe and well tolerated in humans. One unsolved question is whether a complete blockade of baseline C5a levels in humans would impact physiologically relevant processes and therefore be potentially harmful. On the other hand, the duration of antibody administration would likely be limited to days rather than weeks.

Given the available data in the literature as reviewed, C5a activation occurs early in experimental sepsis and significantly contributes to subsequent organ dysfunction and organ failure. C5a thereby appears to be a key player responsible for boosting various immunological inflammatory processes early in sepsis. This makes sense from a biological point of view. In the era before antibiotics, proliferating bacteria in human blood would usually lead to extreme risk of death. Weapons would have to be in place, activating within minutes of infection and amplifying and boosting inflammatory responses with one goal: to get rid of bacterial presence before exponential amplification of the organism(s) occurs. Such would have to occur prior to significant organ damage. With this rationale in mind, it appears reasonable to attempt a rather early or even prophylactic approach for targeting C5a as a harmful key player in sepsis, while keeping up high standards of supportive care and an early focus on eradication of bacteria with antimicrobial therapy. Trial designs will have to allow for administration of a drug targeting C5a. However, until today, little is known about the role of C5a in later phases of sepsis and its postulated contribution to immune paralysis in later phases of sepsis in humans. Trials answering this question will be needed but may be less likely to result in substantial clinical impact for the septic patient who is displaying organ dysfunction or failure.

## 9. The Future

In view of the lack of highly effective drugs for the treatment of human sepsis, the expanding evidence suggests the possible roles of the complement activation product, C5a, and its receptors (C5aR and C5L2) as targets in the highly damaging and lethal consequences of sepsis. Collectively, the data suggest that, in patients with sepsis, use of an antibody that neutralizes C5a but does not cause C5 blockade/depletion may hold clinical promise. The characteristics of the ideal antibody to C5a are continuing to be determined. Clinical trials featuring the use of such an antibody in human sepsis hold considerable promise.

## Figures and Tables

**Table 1:** Survival of k. o. mice after CLP.

Grade of CLP	Genotype	Time after CLP	Survival
High grade*	Wt	Day 3	0%
High grade	C5aR^−/−^	Day 3	85%
High grade	C5L2^−/−^	Day 3	80%
Intermediate grade**	Wt	Day 7	31%
Intermediate grade	C3^−/−^	Day 7	5%
Intermediate grade	C5^−/−^	Day 7	15%
Intermediate grade	C5aR^−/−^	Day 7	80%
Intermediate grade	C5L2^−/−^	Day 7	100%

^∗, ∗∗^Grades as defined in [2, 4].

**Table 2:** Protective effects of anti-C5a rabbit IgG in CLP rats.

Type of antibody	Time for antibody infusion (hr)**	Survival at 7 days*		
		Anti-C5aM	Anti-C5aC	Preimmune IgG
Rabbit IgG (600 *μ*g i.v.)	0	90%	85%	30%
Rabbit IgG (600 *μ*g i.v.)	6	70%	60%	35%
Rabbit IgG (600 *μ*g i.v.)	12	50%	40%	17%

*Expressed as percent of all rats in each group (*n* ≥ 8). The M region of rat C5a involved amino acid residues 17–36 while the C region involved residues 58–77. Peptides were covalently linked to keyhole limpet hemocyanin followed by immunizations of rabbits. Antibodies were isolated by antigen affinity purification. Adapted from data in [13].

**Time (hr) of antibody infusion after CLP.

**Table 3:** Protective effects of mAb to rat C5a*.

Treatment	Amount injected i.v.	Survival (day 5)
Normal IgG_2_	1,000 *μ*g	10%
Anti-rat C5a mAb	1,000 *μ*g	70%
Anti-rat C5a mAb	400 *μ*g	65%

*Monoclonal IgG_2_ obtained by immunization of C57Bl/6 mice with recombinant rat C5a. The antibody was of high affinity based on plasma resonance measurements using a biosensor instrument.

**Table 4:** Activation of caspases in thymocytes after CLP.

Caspase	Treatment*	
	Normal IgG	Anti-C5a IgG
3	3.72	1.75
6	3.0	1.63
8	No change	No change
9	2.85	1.15

*Expressed as fold increase in thymocytes 12 hr after CLP. A fold change of 1.0 would represent no change in caspase activity. Caspase activity was expressed as release (pg/*μ*g protein) of the fluorogenic substrate 7-amino-4-tri-fluoromethyl coumarin (AFC) as described in [20].

**Table 5:** Apoptosis of rat thymocytes 12 hr after CLP*.

Condition	Annexin V binding (% positive thymocytes)	
sham	4%	
CLP + preimmune rabbit IgG (400 *μ*g) CLP + anti-C5a (400 *μ*g)	$\;\begin{array}{c} 29\%\\ 7\% \end{array}\}$	80% reduction

*Thymocytes were obtained 12 hr after CLP and assessed in flow cytometry for binding of Annexin V. Adapted from [20].
